# Lost on the frontline, and lost in the data: COVID-19 deaths among Filipinx healthcare workers in the United States

**DOI:** 10.3389/fpubh.2022.958530

**Published:** 2022-08-25

**Authors:** Loraine A. Escobedo, Brittany N. Morey, Melanie D. Sabado-Liwag, Ninez A. Ponce

**Affiliations:** ^1^Cancer Research Center for Health Equity, Cedars-Sinai Cancer, West Hollywood, CA, United States; ^2^Department of Health, Society, and Behavior, University of California, Irvine, Irvine, CA, United States; ^3^Filipinx/a/o Community Health Association, Los Angeles, CA, United States; ^4^Department of Public Health, California State University, Los Angeles, CA, United States; ^5^UCLA Center for Health Policy Research, Los Angeles, CA, United States; ^6^Department of Health Policy and Management, Fielding School of Public Health, University of California, Los Angeles, Los Angeles, CA, United States

**Keywords:** healthcare workers, Filipino Americans, data disaggregation, COVID-19, mortality

## Abstract

**Background:**

Filipinx Americans working in healthcare are at risk for COVID-19 death but lack consistent mortality data on healthcare worker deaths. The lack of disaggregated data for Asian subgroups proliferates anti-Asian structural racism as the needs of high-risk groups are systematically undetected to merit a proper public health response. We work around this aggregated data problem by examining how the overrepresentation of Filipinxs in healthcare contributes to COVID-19 mortality among Asian American populations.

**Methods:**

To overcome the lack of COVID-19 mortality data among Filipinx American healthcare workers, we merged data from several sources: Kanlungan website (the only known public-facing source of systematically reported mortality data on Filipinx healthcare workers nationally and globally), National Center for Health Statistics, and 2014–2018 American Community Survey. We examined county-level associations using *t*-tests, scatterplots, and linear regression.

**Findings:**

A higher percentage of Filipinxs among Asian Americans was correlated with a higher percentage of COVID-19 decedents who are Asian Americans (*r* = 0.24, *p* = 0.01). The percentage of Filipinx in healthcare remained a strong predictor of COVID-19 deaths among Asian Americans even after adjusting for age, poverty, and population density (coef = 1.0, *p* < 0.001). For every 1% increase in Filipinx among the healthcare workforce, the percentage of Asian American COVID-19 decedents increased by 1%.

**Interpretation:**

Our study shows that the overrepresentation of Filipinxs in healthcare contributes to COVID-19 mortality disparities among Asian Americans. Our findings advocate for systems change by practicing anti-racist data agendas that collect and report on Asian subgroups for effective real-time targeted approaches against health inequities.

## Introduction

Data on COVID-19 consistently show Asian Americans having lower numbers of cases and deaths compared to other major race and ethnic groups in the United States. However, these numbers have not aligned with the reports and experiences coming from diverse Asian American communities, including Filipinx/a/o (hereafter referred to as Filipinx) Americans. Despite calls from advocates and scholars for more disaggregated data that identifies Asian American subgroups in order to illuminate inequities during the COVID-19 pandemic, these data are still consistently lacking in the nation. This research paper uses innovative crowdsourced data in conjunction with detailed race data to work around the lack of disaggregated data to demonstrate how the burden of COVID-19 falling on Filipinx American populations has contributed to Asian American mortality disparities. In the United States, many federal health surveys (e.g., NHANES, BRFSS) have consistently reported that Asian Americans are faring better on most health indicators compared with other major race/ethnicity groups. Asian Americans are least likely to be obese and have hypertension, and have second to the lowest percentage of individuals who have diabetes (NHANES) (1). Asian Americans also have the lowest prevalence of current asthma among children (Health) and cigarette smoking among youth (2). Even when surveys provide more detailed response options for the Asian race question (i.e., NHANES and NHIS), data on Asian Americans are often presented in the aggregate (3). Disaggregated data, however, reveal greater health disparities among Filipinx/a/o (hereafter referred to as Filipinx) Americans compared with other Asian American groups. Filipinxs have higher prevalence of obesity, asthma, and diabetes compared with Vietnamese, Chinese, Japanese and Koreans, and non-Hispanic white (4). Compared to other Asian groups, Filipinxs also have higher prevalence of hypertension compared with non-Hispanic white. Filipinx also had the highest incidence rates of prostate cancer and thyroid cancer, and the highest mortality rates for breast cancer, prostate cancer and thyroid cancer among the largest Asian groups in California (5) but most cancer research continue to report findings in the aggregate (6, 7). These health disparities persist even among Filipinxs who score well on socioeconomic measures that are often associated with better health outcomes (i.e., employment, education, English language proficiency), which suggests the need to expand these traditional measures to include indicators of structural racism that result to entrenched inequities (8–10).

Lack of disaggregated data for Asian American groups supports and proliferates anti-Asian structural racism. Failure to collect and report disaggregated data for Asian American populations is a structural barrier that makes it impossible to describe the COVID-19 burden using unbiased data in these diverse populations. The COVID-19 pandemic has revealed holes in existing public health infrastructure. This includes gaps in our epidemiological data collection and reporting systems, upon which we base our public health programs and policies to mitigate population-level problems such as disparities in disease incidence and mortality. Among United States (US) counties with Asian American populations, the proportion of the population that identifies as Asian American ranges from 0.01% in Red River Parish, Louisiana and Benton County, Mississippi to 43% in Honolulu County, Hawaii. Disaggregation of COVID-19 data for detailed Asian American groups may be hindered by small numbers in counties with statistically insufficient representation of Asian Americans. Data that are lacking in refinement can have dire consequences on populations who become lost or hidden in the statistical reports produced by government agencies. This is certainly true in the US for many minoritized racial or ethnic subgroups who were disproportionately impacted during the COVID-19 pandemic, but whose disparities in infection and death received little attention and no mitigation. One such population are Filipinx Americans, who make up about 18% of Asian Americans (11).

Filipinx Americans, as well as other Asian subgroups, received minimal attention during the COVID-19 pandemic due to the lack of COVID-19 infection and mortality data disaggregated by Asian subgroup in public health surveillance systems. According to the Centers for Disease Control and Prevention, Asian Americans had lower case rates and similar hospitalization and death rates as white Americans throughout the pandemic; this is compared to American Indian or Alaska Native, Black or African American, and Latinx persons, all of whom had higher case rates and over two times the hospitalization and death rates as white persons (12). However, treating Asian Americans as a single aggregate race group in COVID-19 data reporting hides disparities in this diverse population, including among Filipinxs. During one of the peaks of the pandemic on 27 October 2020, restricted death certificate data showed that Filipinxs accounted for 29.5% of COVID-19 deaths among Asian Americans in California, the most of any other Asian subgroup, even though they only represented about a quarter of the state's Asian population (13). Among California's Filipinx healthcare workers, an estimated 6.4% of COVID-19 confirmed cases ended in death, a death-to-case ratio that was nearly 16 times more severe than that of non-Latinx white healthcare workers (14). Filipinxs working in healthcare throughout the US were particularly at risk for COVID-19 death, but lack of consistent data on healthcare worker mortality and data disaggregated by Asian subgroup hampered further examination into this topic. Although Filipinxs make up 4% of the total US nursing workforce, a report from National Nurses United revealed that Filipinxs made up 31.5% of all US nurse fatalities (15). In the US, 16% of nurses are immigrants (16). Filipinxs have long immigrated to the U.S. to fulfill shortages in the healthcare workforce, including in fields such as nursing. The U.S. has had a strong presence in the Philippines for over a century, historically shaping higher education on the Philippines. While many Filipinxs are trained in nursing and related fields, low wages in the Philippines combined with healthcare worker shortages in other countries have led to a brain drain of these workers to countries such as the United States (17–19). Among the foreign-born healthcare workforce in the US, Filipinx immigrants make up 28% of registered nurses, 12% of personal care aides, and 4% of physicians and surgeons (20). In the long-term care sector (home health, skilled nursing facilities, residential care facilities and private household), the percentage of Filipinx healthcare workers is almost 5 times larger than the share of the U.S. general population aged 85 and older who most likely need long-term care services (21).

Filipinxs likely bore a high share of the COVID-19 deaths among Asian Americans in places where they accounted for a large percentage of the population or large proportion of the healthcare workforce. Not only are Filipinxs overrepresented in the healthcare workforce in specific areas round the US, but Filipinxs, also, are more likely than other Asian subpopulations to have preexisting chronic health conditions and live in intergenerational homes, placing the Filipinx community at greater risk of COVID-19 infection and death (4, 22, 23). However, Hawai'i is the only US state that currently disaggregates Asian subgroups in public-facing COVID-19 statistics. There, Filipinxs account for 24% of COVID-19 deaths, although they make up only 16% of the total population (24). The 330 deaths among Filipinxs in Hawai'i as of May 2022 cause them to have the highest COVID-19 death rate (148 per 100,000) in the state among Asian Americans and second only to Pacific Islanders who have the highest COVID-19 death rate. Outside of Hawai'i, data on Filipinx deaths due to COVID-19 disaggregated from the Asian American catch-all are practically non-existent across the nation.

How do we highlight gaps in public health surveillance when the data do not exist? One solution has been for members of the public to crowdsource data. In April 2020, the grassroots transnational organization AF3IRM began the website called “Kanlungan” to memorialize fallen Filipinx/a/o (hereafter referred to as Filipinx) healthcare workers around the world who have died of COVID-19 (25). Kanlungan, the Tagalog word for “refuge” or “shelter,” reflects the AF3IRM's desire to create a space to heal from the loss of so many Filipinxs who were themselves healers by occupation. Kanlungan is the only known source of systematically collected public-facing data on Filipinx healthcare workers deaths in the entire US. The Kanlungan tributes describe Filipinxs, many of whom worked as nurses, doctors, medical technicians, janitorial staff, and other health-related occupations. Currently, the Kanlungan website reports the highest number of fallen Filipinx healthcare workers in the US compared to any other country in the world, including the Philippines. The Kanlungan website provides faces and stories to a narrative where the greatest number of immigrant US healthcare workers who died from COVID-19 are from the Philippines (26).

Salient to exposing hate in Asian American communities, the interlocking relationships between imperialism, transnational labor, and racism on Filipinx healthcare workers contextualizes the production of the health inequities impacting the Filipinx community in the US (17). To further accentuate the point that Filipinxs have contributed to COVID-19 disparities among Asian Americans, we conducted an analysis to evaluate whether the percentage of Filipinxs among the Asian American population and the percentage of Filipinxs among healthcare workers are associated with the percentage of COVID-19 decedents who are Asian Americans in US counties for which data are available.

## Methods

### Data sources

To estimate county of residence of Filipinx healthcare workers who died of COVID-19, we retrieved data from the Kanlungan website during the month of December 2020 (25). In deciding who to include on the website, the AF3IRM team that established the Kanlungan website set two standards in data collection. First, the team found at least one source explicitly stating that the fallen healthcare worker was of Philippine ancestry; this was mostly media articles or obituaries sharing the life stories of the deceased. In a few cases, the confirmation came directly from the deceased healthcare worker's family member who submitted a tribute. Second, the team required a minimum of two sources to identify and announce fallen healthcare workers. We retrieved 86 US tributes from Kanlungan, but only 81 of them had information on county of residence. In total, 45 US counties with at least one reported tribute to a Filipinx healthcare worker who died of COVID-19 were identified for analysis and will hereafter be referred to as “Kanlungan counties.”

Mortality data by county, race, and ethnicity came from the National Center for Health Statistics (NCHS) (27). Updated weekly, this dataset is based on vital statistics data for use in conducting public health surveillance in near real time to provide provisional mortality estimates based on data received and processed by a specified cutoff date, before data are finalized and publicly released (28). We used the data released on 30 December 2020, which included provisional COVID-19 death counts from 1 February 2020 to 26 December 2020—during the height of the pandemic and prior to COVID-19 vaccines being available—for counties with at least 100 total COVID-19 deaths. During this time period, 501 counties (15.9% of the total 3,142 counties in all 50 states and Washington DC) (29) met this criterion. Data on COVID-19 deaths were available for six major racial/ethnic groups: Non-Hispanic White, Non-Hispanic Black, Non-Hispanic Native Hawaiian or Other Pacific Islander, Non-Hispanic American Indian or Alaska Native, Non-Hispanic Asian (hereafter referred to as Asian American), and Hispanic. People with more than one race, and those with unknown race were included in the “Other” category. NCHS suppressed county-level data by race and ethnicity if death counts are <10. In total, 133 US counties reported COVID-19 mortality data for Asian Americans. These data were used to calculate the percentage of all COVID-19 decedents in the county who were Asian American.

We used data from the 2018 American Community Survey (ACS) five-year estimates, downloaded from the Integrated Public Use Microdata Series (IPUMS) to create county-level population demographic variables (30). IPUMS is publicly available, and the database integrates samples using ACS data from 2000 to the present using a high degree of precision (30). We applied survey weights to calculate the following variables at the county-level: median age among Asian Americans, average income to poverty ratio among Asian Americans, the percentage of the county population that is Filipinx, and the percentage of healthcare workers in the county who are Filipinx. Healthcare workers encompassed all healthcare practitioners, technical occupations, and healthcare service occupations, including nurse practitioners, physicians, surgeons, dentists, physical therapists, home health aides, personal care aides, and other medical technicians and healthcare support workers. County-level data were available for 107 out of the 133 counties (80.5%) that had NCHS data on the distribution of COVID-19 deaths among Asian Americans, and 96 counties (72.2%) with Asian American healthcare workforce data.

The ACS 2018 five-year estimates were also the source of county-level percentage of the Asian American population (alone or in combination) who are Filipinx (11). In addition, the ACS provided county-level population counts (29) to calculate population density (people per 1,000 people per square mile), estimated by dividing the total population by the county area, then dividing by 1,000 people. The county area was calculated in ArcGIS 10.7.1 using the county boundary shapefile and projected to Albers equal area conic (for counties in the US contiguous states), Hawai'i Albers Equal Area Conic (for Hawai'i counties), and Alaska Albers Equal Area Conic (for Alaska counties) (23).

### Statistical analysis

For the maps, we obtained county-level Filipinx American population data from the ACS 2018 5-year estimates. We identified counties that belonged to the top quintile of Filipinx American population counts and counties where Filipinx Americans make up more than half of the Asian American population.

We also used the linked data from Kanlungan, NCHS, and ACS to examine county-level characteristics for all counties that reported Asian American COVID-19 mortality data. We compared counties that reported at least one Kanlungan death (i.e., Kanlungan counties) to counties that did not report any Kanlungan death (i.e., non-Kanlungan counties). We used independent *t*-tests for two samples to determine the *p*-values for differences between Kanlungan counties and non-Kanlungan counties. Next, we determined whether the county-level percentage of Asian Americans among COVID-19 deaths was correlated separately with county-level percentage of Asian Americans who are Filipinx and percentage of healthcare workers who are Filipinx.

We constructed simple and multivariable linear regression models separately for the two main independent variables—percentage of population who are Filipinx and percentage of healthcare workers who are Filipinx—to assess how each is associated with the percentage of COVID-19 decedents who are Asian Americans. Multivariable stepwise forward regression models accounted for median age, household income to poverty ratio, and population density to rule out potential confounding due to these county-level factors. These variables were chosen as potential confounders, since they might be independently associated with both the Filipinx population as well as COVID-19 deaths. We restricted the models to counties with information on all the covariates.

## Results

**Figure 1:**
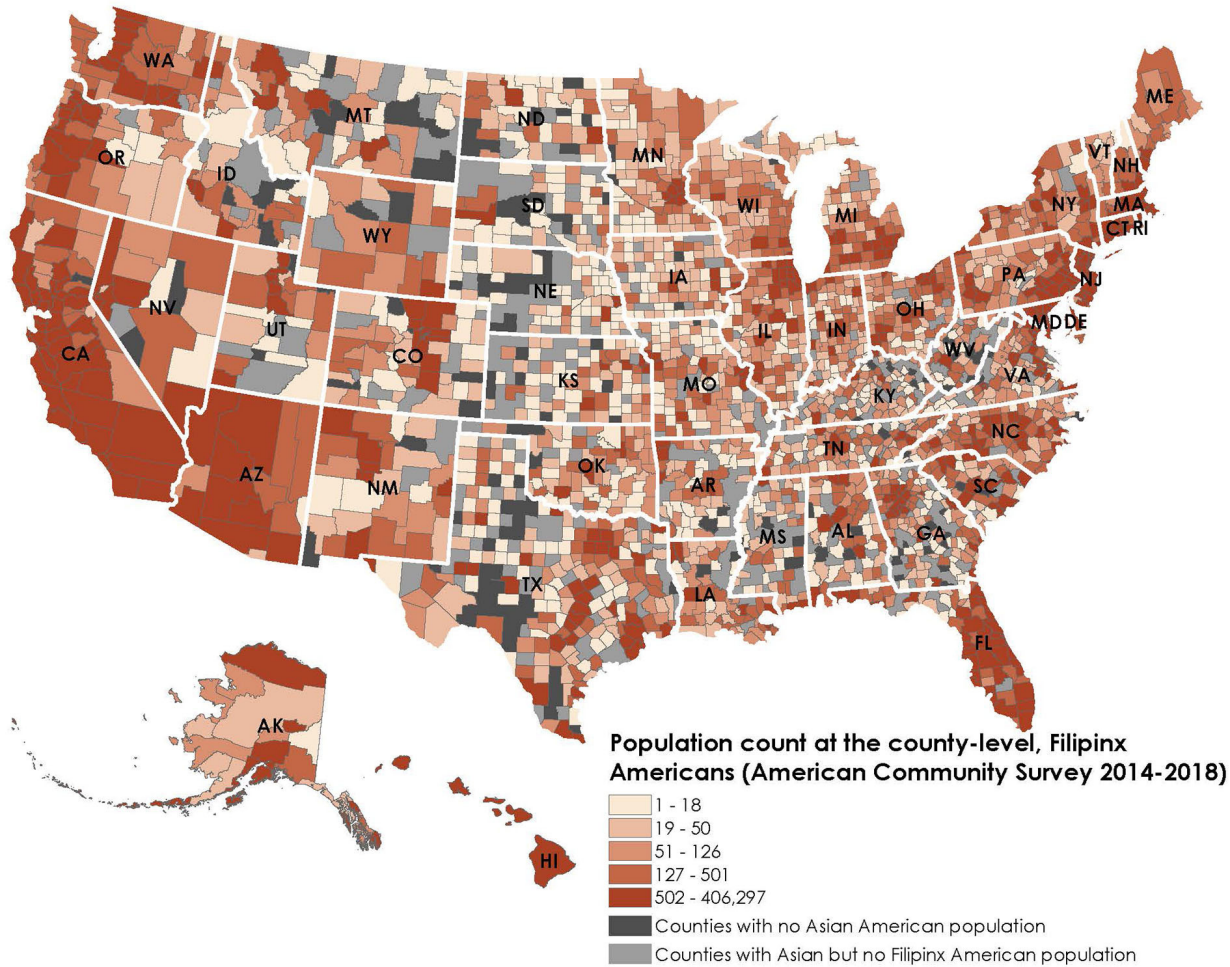
Population count, Filipinx Americans, county-level, 2014–2018.

Figure 1 shows that 505 counties belonged to the top quintile of Filipinx American population count across 49 states and Washington D.C. (except Vermont). In the top twenty counties with the largest Filipinx American population, Filipinx American population count ranged from 47,350 (Margin of Error: 2,528) to 406,297 (Margin of Error: 6,250)(counties of Los Angeles, Honolulu, San Diego, Clark, Santa Clara, Alameda, Orange, San Mateo, Cook, Riverside, King, Contra Costa, Sacramento, San Bernardino, Solano, San Joaquin, Maui, Maricopa, San Francisco and Queens). Filipinx Americans live in 85.3% of counties that have Asian American populations, compared to 75.0% for Chinese Americans and 68.1% for Asian Indian Americans.

**Figure 2:**
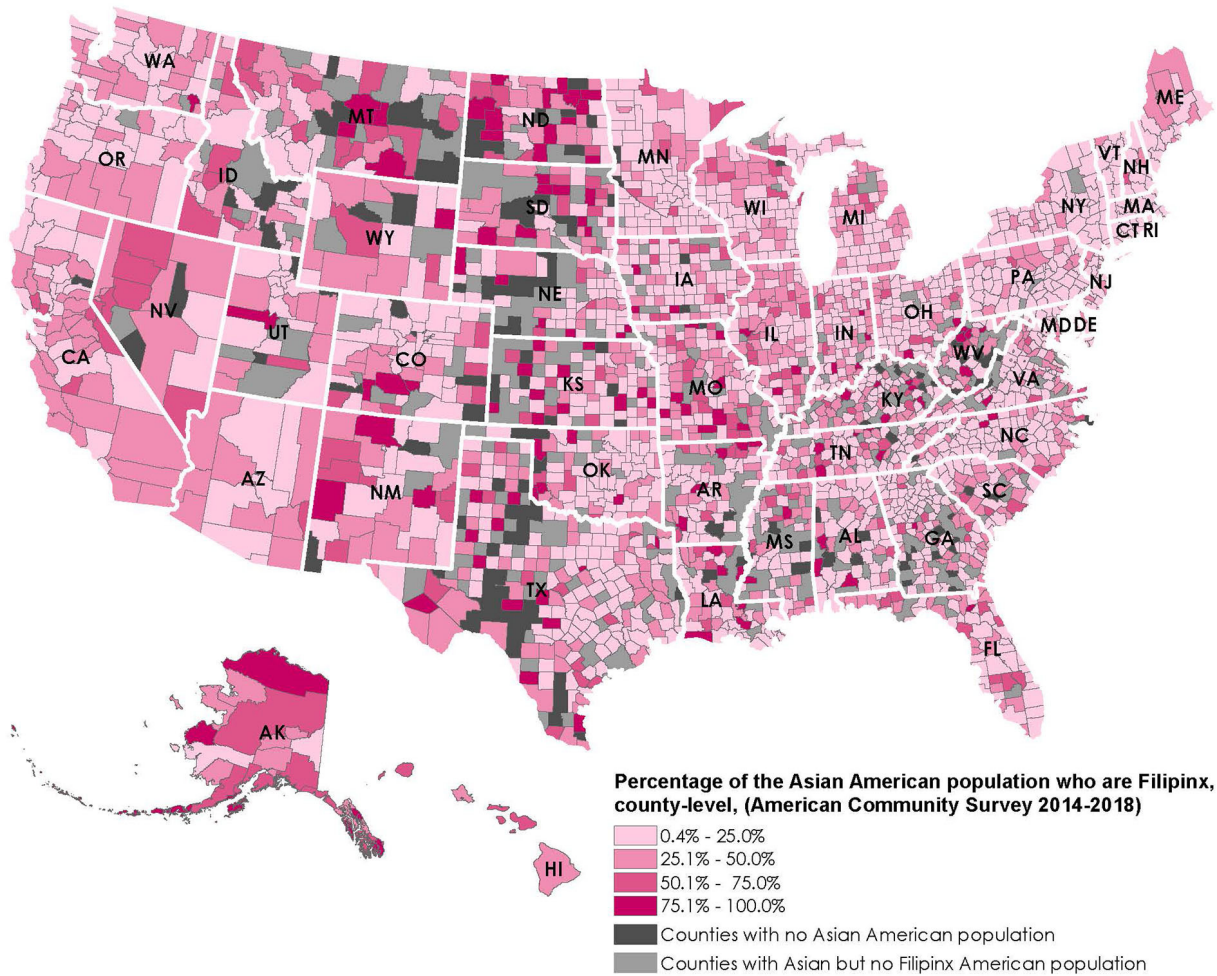
Percentage of the Asian American population who are Filipinx, county-level, 2014–2018.

Meanwhile, Figure 2 shows that Filipinx Americans make up more than half of the Asian American population in 355 counties across 40 states. Among 1,483 rural counties with the smallest Asian American populations (i.e., the lowest two quintiles of Asian American population ranging from 1 to 229), Filipinx Americans make up more than half of the Asian population in 278 counties. They are present in more rural counties than the two largest Asian groups in the US: Chinese Americans are present in 119 and Asian Indian Americans are present in 117 of these rural counties.

Table 1 shows summary statistics for all 133 US counties included in the analysis. On average, the median age among Asian Americans was 34 years old and the mean household income to poverty ratio among Asian Americans was 5.5. Asian Americans made up 8.8% of the total population on average in these counties, and Filipinxs made up 15.3% of the Asian population on average in these counties. Filipinx account for an average of 4.7% of the healthcare work force in these counties. Among COVID-19 decedents, 6.1% on average were Asian Americans. Median age of the Asian American population and household income to poverty ratio were slightly higher in Kanlungan counties compared to non-Kanlungan counties, although the *p*-values did not fall below 0.05 (*p* = 0.09 for median age and *p* = 0.49 for household income to poverty ratio). The mean population density in Kanlungan counties (7.3 population per 1,000 population per square mile) was much higher than in non-Kanlungan counties (1.9 population per 1,000 population per square mile) (*p* = *0*.04). Compared to non-Kanlungan counties, the mean percentage of the population who are Asian Americans, the mean percentage of Asian Americans who are Fillipinx, and the mean percentage of the healthcare workforce who were Filipinx are higher in Kanlungan counties, but none of the p-values for these differences fell below 0.1. In Kanlungan counties, 7.0% of COVID-19 decedents were Asian Americans which was slightly higher than in non-Kanlungan counties (5.8%), but the difference did not reach statistical significance (*p* = 0.38).

**Table 1:** Summary statistics for U.S. Counties that reported COVID-19 deaths among Asian Americans.

**County-level variable**	**All counties (*n* = 133)**	**Kanlungan counties^a^** **(*n* = 45)**	**Non-Kanlungan counties^b^** **(*n* = 98)**	***p*-value^c^**
	**Mean (SD)**	**Mean (SD)**	**Mean**	
Median age among Asian Americans	33.6 (3.3)	34.4 (2.8)	33.2 (3.5)	0.09
Mean household income to poverty ratio among Asian Americans	5.5 (1.5)	5.7 (1.5)	5.4 (1.4)	0.49
Population density^d^	3.3 (7.9)	7.3 (14.4)	1.9 (2.6)	**0.04**
Percentage of population who are Asian Americans^e^	8.8 (7.3)	10.3 (7.7)	8.3 (7.2)	0.17
Asian Americans who are Filipinxs	15.4 (11.2)	18.7 (11.2)	14.3 (11.0)	**0.04**
Healthcare workforce that is Filipinx	4.7 (5.3)	6.1 (4.9)	4.1 (5.4)	0.08
COVID-19 decedents who are Asian Americans^e^	6.1 (6.9)	7.0 (5.9)	5.8 (7.2)	0.38

a Counties with at least one tribute to a Filipinx healthcare worker who died of COVID-19 reported in the Kanlungan website.

b Counties that have no reported tributes to a Filipinx healthcare worker who died of COVID-19 in the Kanlungan website.

c P-values are from the independent t-tests for two samples comparing counties where one or more Filipinx healthcare worker death is reported on the Kanlungan website to counties where no Filipinx healthcare worker deaths are reported on the Kanlungan website. We used the Satterthwaite variance estimator if the variances for the two groups are statistically different, and the pooled variance estimator if the two groups have the same variance.

d Population density is measured as people per 1,000 people per square mile.

e Asian Americans refer to non-Hispanic Asians.

Linear correlation analyses show that both the percentage of Filipinx among Asian Americans (*r* = 0.24, *p* = 0.01) and percentage of Filipinx in the healthcare workforce (*r* = 0.73, *p* <0·0001) were positively correlated with percentage of COVID-19 decedents who are Asian Americans, but the latter correlation was higher. Table 2 shows the results of the simple and multivariable linear regression models estimating the association between the county-level percentage of Asian Americans who are Filipinx and the percentage of COVID-19 decedents who are Asian Americans. An increase in the Filipinx percentage of the Asian American population in the county was associated with a higher percentage of COVID-19 decedents who are Asian Americans (Model 1: coef = 0.2, *p* < 0.05) but the positive linear association was attenuated after adjusting for median age (Model 2: coef = 0.1, *p* > 0.05). The association remained minimal after adjusting for household income to poverty ratio and population density in Models 3 and 4. Table 3 shows a positive linear association between the percentage of healthcare workers who are Filipinx and the percentage of COVID-19 decedents who are Asian Americans (Model 1: coef = 1.0, *p* < 0.001). This positive linear association remained after accounting for median age, household income to poverty ratio, and population density. The results suggest that the percentage of COVID-19 decedents who are Asian Americans increases by 1.0% (standard error: 0.1) on average for every 1.0% increase in the percentage of healthcare workers who are Filipinx, after accounting for median age, household income to poverty ratio, and population density (Model 4).

**Table 2:** Regression of percentage of COVID-19 decedent who are Asian Americans on percentage of Asian Americans who are Filipinx in U.S. counties, *n* = 107.

**Variables**	**Model 1**	**Model 2**	**Model 3**	**Model 4**
Percentage of Asian Americans who are Filipinx	0.2* (0.1)	0.1 (0.1)	0.04 (0.1)	0.1 (0.1)
Median age among Asian Americans		1.0** (0.2)	1.1** (0.2)	1.1** (0.3)
Mean household income to poverty ratio among Asian Americans			−0.4 (0.5)	−0.4 (0.5)
Population density (per sq. mi.)				0.1 (0.1)
Constant	4.1* (1.2)	−27.3** (6.6)	−28.5** (6.8)	−27.7** (6.9)
Adjusted R–squared	0.05	0.21	0.21	0.21

Standard errors are reported in parentheses. *p < 0.05, **p < 0.001.

**Table 3:** Regression of percentage of COVID-19 decedents who are Asian Americans on percentage of healthcare workforce that is Filipinx in U.S. counties, *n* = 96.

**Variables**	**Model 1**	**Model 2**	**Model 3**	**Model 4**
Percentage of healthcare workforce that is Filipinx	1.0** (0.1)	0.9** (0.1)	0.9** (0.1)	1.0** (0.1)
Median age among Asian Americans		0.2 (0.2)	0.3 (0.2)	0.2 (0.2)
Mean household income to poverty ratio among Asian Americans			−0.2 (0.4)	−0.2 (0.4)
Population density (per sq. mi.)				0.1* (0.1)
Constant	1.8* (0.7)	−5.6 (5.7)	−6.0 (5.8)	−4.3 (5.8)
Adjusted R–squared	0.52	0.53	0.52	0.55

Standard errors are reported in parentheses. *p < 0.05, **p < 0.001.

## Discussion

The COVID-19 pandemic revealed serious and detrimental gaps in public health surveillance by racial and ethnic subgroups, including for Filipinx Americans. We contend that lack of detailed surveillance data for Asian subgroups is a form of anti-Asian structural racism that causes disparities to be invisible and solutions to be non-existent.

### Aggregated data workaround

Evidence shows that Filipinxs have died at a disproportionate rate due to COVID-19, but the lack of data makes this disparity lost in the vast majority of data reporting. Crowdsourced data including the Kanlungan tribute and reports of high rates of COVID-19 death among Filipinx healthcare workers, have attempted to fill this data void. In addition, to work around the lack of disaggregated data, we used county-level population data to show the strong correlations between the percentage of Filipinxs among Asians and the percentage of Filipinxs among healthcare workers with Asian American COVID-19 mortality. These data workarounds together provide further evidence of COVID-19 mortality disparities for Filipinxs, evidence that should illicit public health action. However, these data solutions are far from perfect but a consequence of this “data tax” on populations invisible in public health data systems. More consistent and widespread disaggregation of data are needed to highlight the current COVID-19 disparities, as well as other disparities that Filipinxs and other Asian subgroups continue to experience.

### Data justice for Filipinx American healthcare workers

The overrepresentation of Filipinxs in deaths among healthcare workers is indicative of inadequate workplace precautions meant to shield healthcare workers from exposure to patients with suspected or confirmed COVID-19 within the first year of the pandemic (31). This inadequacy of workplace protections may be shaped by what Nazareno and colleagues (25) note as “socially constructed power inequities and inferior views of Filipinos” owing to the past US imperial presence in the Philippines. The unique past colonial relationship between the US and the Philippines (17, 32) may still be embedded in contemporary workplace dynamics manifesting in suppression of agency to request Personal Protective Equipment (PPE), and fueling social desirability of proving worth by volunteering to be the first to take care of the sickest COVID-19 patients at the cost of self- and family-care, mentally and physically. Certainly, this was the narrative heard from media coverage and discussed extensively in recent conceptual and empirical work (25) that estimated higher proportions of Philippine-trained nurses working in inpatient care and acute care settings than US-trained nurses.

Our findings reveal higher percentage of Filipinx Americans among Asian Americans was correlated with a higher percentage of COVID-19 decedents who are Asian. Moreover, the percentage of Filipinx in healthcare was a strong predictor of COVID-19 deaths among Asian Americans in those counties even after adjusting for age, poverty, and population density. Furthermore, the contribution of the percentage of the healthcare workforce that is Filipinx to overall Asian American COVID-19 mortality suggest that not only are Filipinx healthcare workers are at high risk of mortality with infection, but also their high-risk occupations may unintentionally be contributing to the spread of COVID-19 to their family members who do not work in healthcare. Risk to older household members could be higher in Filipinx families. Compared to the US population (19%), a higher share of Asian Americans live in intergenerational households (26%), (26) and Filipinxs (33%) have a higher percentage of intergenerational households than Asian Americans overall (33).

Counties with a high percentage of healthcare workers who are at increased risk should prioritize reducing the number of COVID-19 infections among their healthcare workforce by tracking infections, reducing spread, and enhancing workplace safety protocols if needed (34). This also means providing appropriate contact tracing, resources, and healthcare for infected healthcare workers to recover and to be able to protect their households from infection. Social determinants of health and occupation-related factors should be considered together with underlying chronic conditions when designing public health strategies that aim to reduce the COVID-19 mortality burden among the most impacted populations (29). Furthermore, these healthcare workers made high-risk by working on the frontline in direct care and support occupations (i.e., housekeeping staff) must be prioritized by leadership for burnout and mental health distress prevention and support among their staff, specifically in acute/critical care settings, outpatient and long term care facilities, as well as skilled nurse facilities, hospice care, and home healthcare settings. Such plans to support healthcare staff may also consider normalizing policies that support families and household members who are in close contact with frontline workers on a daily or regular basis. This may include resources that help families to understand the extent of and provide support for healthcare worker burnout, mental health challenges, or traumatic experiences compounded by the pandemic.

Disaggregated race data for Asian subgroups is absolutely vital to addressing disparities. Unfortunately, underlying systematic barriers in the form of low funding rate for health research focused on Asian Americans and for supporting the careers of Asian American researchers derail any progress to date. In the past two decades, only 0.17% of the overall National Institutes of Health (NIH) budget was allocated to Asian American, Native Hawaiian, and Pacific Islander (AA/NHPI)-related clinical research projects (35). Of these projects, only 60% mentioned an AA/NHPI subgroup. Only 5% of the total funding allocated to AA/NHPI-related projects were for research career awards, training grants, and fellowships. To date, only the state of Hawaii publicly reports data on cases and deaths for their most populous Asian subgroups, which includes Filipinx Americans. Disaggregated COVID-19 mortality data in California was only available as restricted data; and the inclusion of Filipinxs in the state's COVID-19 vaccination plan was a modest acknowledgment of the disproportionate impact on the Filipinx community (30). The common practice of lumping all Asian Americans together perpetuates the racist trope that all Asians are alike and lends to the invisibility of distinct Asian communities (31). Filipinx are the third largest Asian American subgroup in the US, after Chinese Americans and Asian Indian Americans who more likely live in discernible ethnic enclaves throughout the US. Unlike their counterparts, the Filipinx population are largely scattered within metropolitan neighborhoods/cities and across rural spaces to fill in healthcare and teaching gaps (32). Our figure visualizes that Filipinxs live in 85.3% of counties that have Asian American populations, compared to 75.0% for Chinese Americans and 68.1% for Asian Indian Americans (11). Filipinxs also make up the predominant Asian subgroup in 34.5% of counties where Asian Americans live and where there is a predominant Asian subgroup, compared to 12.9% for Chinese Americans and 11.4% for Asian Indian Americans (11). Therefore, we recommend that states such as California, New York, Texas, New Jersey, Illinois, Washington, Florida and Virginia—where the largest Asian populations are located (36), even larger than Hawai'i—consider disaggregating Asian American data for the largest Asian subgroups. Other states such as Nevada, Alaska, Virginia, Maryland, and Massachusetts, where Asian Americans make up a larger than the US average (6.7%) proportion of the state's population (36), are highly encouraged in doing the same. Community groups have also been advocating for county-level disaggregated data for Asian subgroups. In Clark County, Nevada (which includes all the residents surrounding Las Vegas) for example, a majority (51.5%) of Asians Americans are Filipinxs (11) but, to date, the health district's public-facing COVID-19 aggregates Asian all together and combines them with Pacific Islanders as well, hiding potential disparities (37).

### Future considerations

Improved data reporting on mortality and morbidity among healthcare workers would also help to address the toll that the COVID-19 pandemic and future public health emergencies have. A rapid expert consultation from the National Academies of Sciences, Engineering and Medicine concluded that a robust national data reporting system would support strategies and policies to reduce COVID-19 mortality and morbidity among healthcare workers (38). For this data system to accurately capture the disproportionate toll on race/ethnic minority populations, it should include requirements to report data for race/ethnicity groups that are more detailed than the standards set forth by the Office of Management and Budget (35). Meanwhile, given our findings, we recommend that the top counties where Filipinxs make up a large percentage of the healthcare workforce, including the counties of Alameda, Contra Costa, Los Angeles, San Diego, San Joaquin, San Mateo, Santa Clara, and areas surrounding metropolitan counties like San Bernardino and Riverside (all counties are in California), Honolulu (Hawai'i), Clark (Nevada), Bergen and Hudson (both in New Jersey) where more than 10% of the healthcare workforce are Filipinx (30), should at least disaggregate Filipinxs from Asian Americans in not only COVID-19 incidence and mortality data, but public health surveillance of beyond the traditional health indicators (e.g., health insurance, education, income). Of course, confidentiality and stability of data are of utmost importance, and data producers should continue to test various suppression and significance rules in different geographies, in order to provide meaningful disaggregated data that could inform public health policy. Disaggregated Filipinx data for healthcare workers and the population at large are needed in real-time to diagnose issues of structural racism and systemic discrimination that plagues proper resource allocation, leading to disproportionate health outcomes by race and other forms of oppression (e.g., gender, disability, immigration status), which are prominent and pronounced during public health emergencies and crises.

Furthermore, workplace protections must be put into place to protect Filipinx healthcare workers in clinical settings. This can include providing adequate personal protective equipment (PPE), as well as having safety and mitigation policies in place to protect health and well-being,. These policies may ensure sure that healthcare workers feel supported and have adequate time to perform their duties while protecting their own health and the health of their patients. High quality continuing education paid by employers should include leadership training that communicates the appropriate allocation and use of PPE as well as tips to support self-care and prevent burnout during times of high patient census and crises events. We further emphasize the importance of providing Filipinx healthcare workers with high-quality healthcare and medical leave, not only for themselves, but also to care for their immediate and extended family members beyond the allocated government provision of “COVID hours” (i.e., supplemental paid sick leave) during the pandemic. As Filipinx are more likely to live and interact in close-proximity with others (i.e. intergenerational household, multi-family homes), it is important to protect the well-being of Filipinx healthcare worker households as a means of further ensuring workplace retention and positive health outcomes. It is quite possible that Filipinx healthcare workers are being culturally exploited, undervalued, and underpaid for their labor (32). Adequate pay and benefits are essential social determinants of health for Filipinx healthcare workers that can lessen psychological distress and prevent or delay chronic health issues. Lastly, evidence shows that Filipinx healthcare workers experienced discrimination while on the job during the COVID-19 pandemic (39). Filipinxs who experience more workplace discrimination have higher number of health conditions, which can lead to greater mortality (40). Hospital and healthcare settings should mandate anti-discrimination and anti-bias trainings and policies to lessen the additional discrimination stress in the workplace environment.

### Limitations

There are a few limitations that should be mentioned. First, our analysis did not account for county-level variation in local stay-at-home orders and guidelines for reopening, but in supplemental analysis (data not shown) using a state-fixed effects unadjusted model, the magnitude of our point estimates for the Filipinx healthcare workers model was comparable and continued to be significant. Second, Kanlungan staff adopted clear and consistent inclusion criteria to include in the list of published tributes but search terms on explicit mentions of terms such as “birthplace” and “Philippines” may have biased the sample toward immigrant Filipinos. Additionally, reliance on specific social networks, media sources, and online obituaries may have led to selection bias and, as a result, many more Filipinx deaths among healthcare workers may have been left out. Therefore, the total count of COVID-19 deaths among Filipinx healthcare workers and total count of Kanlungan counties could have been underestimated. It is expected that the number of Filipinx COVID-19 deaths in the Kanlungan data (*n* = 86) would be higher than the count reported by National Nurses United (*n* = 67) because the former included three more months of data and non-registered nurses (15). Despite this limitation, we believe Kanlungan is the best source of geographic data on COVID-19 deaths among Filipinx healthcare workers publicly available at this time. Third, the NCHS mortality data used in this study are based on provisional estimates, and are therefore, incomplete due to the time lag between when the death occurred and when the information on the death certificate becomes available for NCHS mortality surveillance, which is also dependent on the surveillance protocols and personnel in place which may have caused further delay during the pandemic (28). Fourth, underlying medical comorbidities that increase the severity of COVID-19 (e.g. asthma, cancer, heart conditions, diabetes etc.) were not included in the models because they were unavailable for Asian Americans at the county level. If data are available, future studies may wish to investigate the extent to which comorbidities among Filipinx contributed to COVID-19 mortality longitudinally. Fifth, margins of error (MOE) and the coefficients of variation (CV) that could inform the reliability of the population estimates from the American Community Survey were not reported. The choice of a CV cutpoint that defines an acceptable range of error depends on the analysis, and more work is needed to determine if there are CV thresholds appropriate for a specific population, geography or application. Moreover, the call for more disaggregated data should be paired with a call for innovative methods and data visualization that address potentially large sampling errors and confidence intervals associated with smaller subpopulations. Our findings suggest that even with its limitations and small sample size, data on Filipinx healthcare worker deaths from the Kanlungan website tracked well with both the percentage of Filipinxs among healthcare workers and the percentage of COVID-19 decedents who were Asian American. The Kanlungan website provided rapid early warning signs and justification for examining the contribution of Filipinx healthcare workers to COVID-19 mortality among Asian Americans. Our study clearly shows that this overrepresentation of Filipinxs in healthcare contributes to COVID-19 mortality disparities among Asian Americans.

Addressing gaps in data that would quantify morbidity and mortality burden among Filipinxs, healthcare workers, and other understudied US populations would result to more targeted and real-time solutions that mitigate risk factors for serious complications associated with COVID-19 and other emerging health problems. Data disaggregation that takes into account the intersectionality of identities experienced by vulnerable populations should also be considered (41). Providing the public with timely death data stratified by, for example, both race/ethnicity and occupation, or by occupation and urban-rural areas (information that are already available in vital statistics or may easily be linked), may produce public health and public policy interventions that could stave off the disturbingly wide health disparities that continue to progress, as was highly apparent during the height of COVID-19 pandemic in late 2020 and in early 2021. These efforts will help to build a better public health infrastructure to ensure that in future events, people who are being lost on the frontline during public health emergencies are not also being lost in reportable mortality data (15).

## Data availability statement

Publicly available datasets were analyzed in this study. This data can be found at: https://doi.org/10.6084/m9.figshare.20353368.v1.

## Ethics statement

Ethical review and approval was not required for the study on human participants in accordance with the local legislation and institutional requirements. Written informed consent for participation was not required for this study in accordance with the national legislation and the institutional requirements.

## Author contributions

LE completed the data analyses and interpreted the data. BM performed the literature review and assisted in pulling data from sources. MS-L assisted in literature review and formatting and submission. NP helped edit the research questions, verified the underlying data and analyses, and guided overall execution. All authors participated in the conceptualization, research, drafting, editing of the manuscript, had full access to the full data used in the study, and accept responsibility to submit for publication. All authors contributed to the article and approved the submitted version.

## Funding

NP's work on this study was partially supported by the Robert Wood Johnson Foundation grant #76329. BM received funding from the UC Irvine Institute for Clinical and Translational Science, Pilot Study Award. MS-L reports support from the NIMHD/NIH RADx-UP (U01MD017434, mPI: MS-L and Kwan). These funders had no role in the study design, data analysis, data interpretation, or writing of the report. All authors had full access to the full data used in the study and accept responsibility to submit for publication.

## Conflict of interest

The authors declare that the research was conducted in the absence of any commercial or financial relationships that could be construed as a potential conflict of interest. The handling editor LÐ declared a past co-authorship with the author(s) NP and BM.

## Publisher's note

All claims expressed in this article are solely those of the authors and do not necessarily represent those of their affiliated organizations, or those of the publisher, the editors and the reviewers. Any product that may be evaluated in this article, or claim that may be made by its manufacturer, is not guaranteed or endorsed by the publisher.
